# Reply to Smith et al.: Social tipping dynamics in a world constrained by conflicting interests

**DOI:** 10.1073/pnas.2002648117

**Published:** 2020-04-23

**Authors:** Ilona M. Otto, Jonathan F. Donges, Wolfgang Lucht, Hans Joachim Schellnhuber

**Affiliations:** ^a^Earth System Analysis, Potsdam Institute for Climate Impact Research, Member of the Leibniz Association, 14473 Potsdam, Germany;; ^b^Stockholm Resilience Centre, Stockholm University, 11419 Stockholm, Sweden;; ^c^Department of Geography, Humboldt University, 10099 Berlin, Germany;; ^d^Integrative Research Institute on Transformations of Human-Environment Systems, Humboldt University, 10099 Berlin, Germany;; ^e^Department of Earth System Science, School of Science, Tsinghua University, Beijing 100084, People’s Republic of China

We fully agree that, in analyzing social tipping interventions (STIs) for accelerating a global transformation to carbon neutrality by 2050 (1), there is a need to analyze social change processes and social movements in greater depth (2). We hope that more research will follow and each of the identified STIs and the key actors that have agency (3) to intervene and initiate such processes will be critically assessed by the research community. The proposed STIs do not encompass only governmental actors. We argue that a radical change can be initiated by a spectrum of actors, including for example financial investors, homeowners, business managers, and educators, all operating on different social structural levels. We welcome the suggestion to investigate use of a complex contagion model to include effects of local clusters of strong ties and their potentially amplifying interactions across social networks, as well as effects of other mesoscopic and macroscopic patterns in network structure (4). However, more empirical data and progress in the formalization and generalization of interconnections seen in case studies is needed to fully assess these complex contagion processes (5).

We also fully agree that coalitions of people and organizations are needed that mobilize around a common cause, eventually reach a critical mass, and produce a momentum for change that in turn legitimizes politicians to build their own coalitions that lead to government action. Indeed, as Otto et al. (1) argue, the last 30 y of international climate action suggest that national governments have turned out to be largely ineffective in solving the climate crisis, despite a relatively high social recognition of climate change as a serious problem. For example, in most European countries, more than 90% of representative survey respondents think that the world’s climate is changing, more than 90% of the survey respondents concur with science that climate change is caused by human activity, and more than 70% of the respondents believe that climate change impacts will be bad (6). Even in the United States, most of the representative survey participants believe that global warming is caused by human activities (64%) and that its effects have begun (60%) (7).The recent Fridays4Future global youth movement demonstrates the massive potential of social movements for climate action. The question is how to translate the public sentiment (or even fear and anger) into political action in a world constrained by conflicting interests (8). This calls for a stronger uptake of both established and novel theories of social change in the decarbonization and Earth system stabilization debates. They could demonstrate how societies might react to political realities where politicians are often resistant to change, tied up by lobby groups and other power structures, do not look at timescales beyond the election period, or use lies, misinformation, and populist arguments to get to power. The scientific community should seek to understand under which conditions a social movement can be converted into crucial legislation.
